# Laparoscopic Gastric Gastrointestinal Stromal Tumor (GIST) Resection in a 108-Year-Old Patient

**DOI:** 10.7759/cureus.81146

**Published:** 2025-03-25

**Authors:** Saima Islam, Thomas A Eldredge, Christophe R Berney

**Affiliations:** 1 Surgery, Bankstown Hospital, Sydney, AUS; 2 Upper GI, Bankstown Hospital, Sydney, AUS

**Keywords:** centenarian, gastrointestinal stromal tumor (gist), multidisciplinary decision-making, operative intervention in geriatric patient, peri-operative care

## Abstract

Surgery for centenarians comes with unprecedented challenges and risks. The limited physical reserve and fragility of homeostatic control in this population reduces their ability to adapt to physiological changes encountered during open and laparoscopic surgery. This case report presents a 108-year-old female patient who underwent laparoscopic resection of a symptomatic gastric gastrointestinal stromal tumour (GIST), without any adverse effects. The case highlights the importance of thorough preoperative risk assessment and planning, multidisciplinary input, and individualised patient care.

## Introduction

Life expectancy in most developed countries continues to rise, increasing by 4.8 years for males and 3.2 years for females over the past two decades in Australia [1]. In line with this, more patients above the age of 90 years are undergoing both elective and emergency surgery [2]. This cohort typically has significant medical comorbidities and limitations in functional ability, which can be anticipated to impact their recovery outcomes. The reported and estimated mortality rate for any hospitalisation in centenarians is greater than 10% [3]. Published outcomes of general surgical procedures in centenarians are scarce in the scientific literature.

Selecting appropriate surgical candidates among the elderly is fraught with challenges, primarily due to the combination of reduced physiological reserve, higher risk of complications and the presence of multiple comorbid conditions. These complexities often result in under-treatment of localised gastrointestinal stromal tumour (GISTs) in older patients, with elderly individuals receiving fewer surgical interventions and adjuvant treatments, ultimately leading to poorer outcomes [4].

While age has traditionally been recognized as a risk factor for perioperative morbidity and mortality, studies suggest that age alone does not predict postoperative complications following elective surgery in older patients, instead comorbidity burden and frailty maybe more important predictors of postoperative outcomes in this population [5]. As such, individuals aged 100 years and older who have operable diseases should not be denied surgical intervention due to perceived risks associated with advanced age [6].

Here, we report the case of a 108-year-old patient who successfully underwent laparoscopic resection of a gastric gastrointestinal stromal tumour (GIST), presenting with symptoms of chronic abdominal pain, anaemia, and gradual weight loss.

This case highlights the potential for successful outcomes in centenarians, particularly with minimally invasive surgery and comprehensive, individualized care. The positive outcome underscores the feasibility of minimally invasive surgical interventions in this patient group and provides insight into the importance of an optimised perioperative care, multidisciplinary approach in geriatric surgery.

## Case presentation

A 108-year-old female patient, born in 1915, presented with chronic nonspecific upper abdominal pain and 10-kilogram weight loss over a period of three years, associated with progressive iron deficiency anaemia. Her pre-morbid level of function was good; she lived at home with her daughter and son, remained independent with her personal daily activities and mobilised with a walking stick. She had support from family and community service when needed. Her past medical history included ischaemic heart disease, previous cerebrovascular accident with some minor residual left-sided weakness, type 2 diabetes mellitus, mild dementia, hearing impairment, and glaucoma.

**Figure 1:**
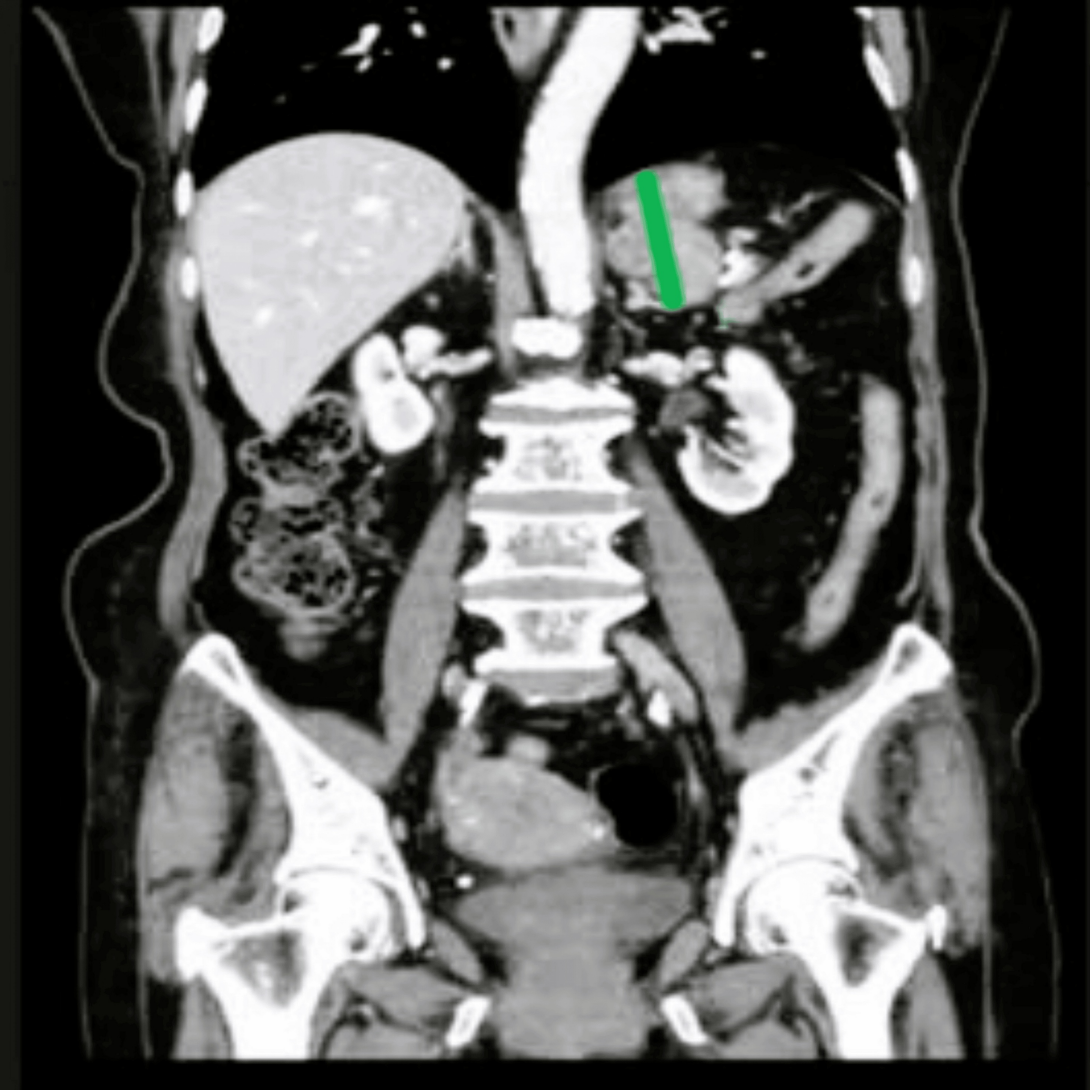
Lobulated heterogeneously enhancing mass arising exophytically from gastric fundus measuring 45x34 mm axially and 45 mm craniocaudally (represented by the green line)

Initial workup with computed tomography revealed an exophytic mass in the posterior gastric fundus (Figure 1). The lesion was favoured to be a GIST, with resection a viable option. Extensive pre-operative consultation between the surgeon, anaesthetist, patient and her children was undertaken before proceeding with surgery. The primary indication was to alleviate the pain and prevent any bleeding/perforation from the tumour. A comprehensive individualised perioperative management plan was devised, incorporating medical and nursing staff, allied health and community services.

Pre-operative management

The pre-operative planning involved consultation with various specialists, including anaesthetist, surgeon, cardiologist, and intensivist. The aim was to assess her fitness for surgery and minimise potential risks. The anaesthetist performed a detailed pre-operative assessment, considering her history of ischemic heart disease, type 2 diabetes, and previous cerebrovascular accident. Routine investigations were reviewed and no acute abnormalities detected. A plan was made for the patient to be closely monitored in a high-dependency unit (HDU) after surgery. A tailored anaesthesia plan was developed to ensure stability during the procedure. Her medications were carefully rationalised; her regular oral hypoglycaemic agents were temporarily withheld to prevent metabolic complications, and aspirin was also temporarily discontinued one week pre-operatively to minimise the risk of perioperative bleeding. Given her advanced age and cognitive status, her family was actively engaged in decision-making, ensuring that the patient’s preferences and quality of life were prioritized.

Surgical intervention

She underwent laparoscopic resection (partial fundectomy) of the mass in August 2023. No mucosal abnormality or mass was visible on intraoperative gastroscopy. The procedure was carefully planned to minimise operative time and blood loss, considering her advanced age and potential for poor healing. General anaesthesia was required for laparoscopy, and multimodal analgesia was utilised to minimise opiate use.

**Figure 2:**
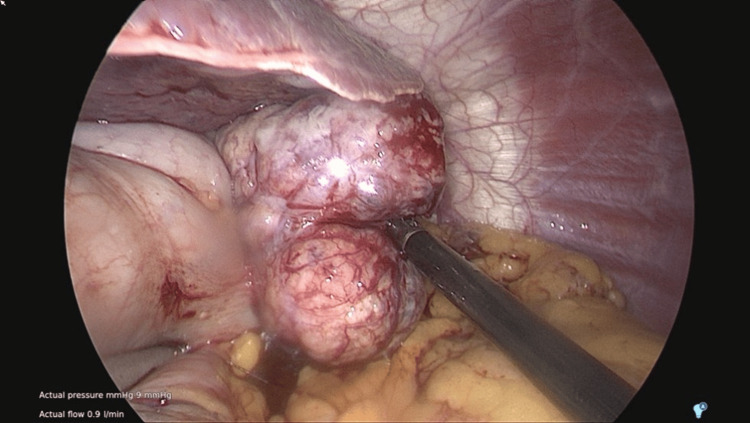
Intraoperative photo: Tumour located over gastric fundus

Intra-operatively, pneumoperitoneum pressure of 8mmHg was used to minimise the impact on venous return. The procedure was completed using two 12mm and two 5mm access ports. A stapled resection of the gastric fundus with clear macroscopic margins was achieved after appropriate mobilization (Figure 2). The specimen was extracted through the umbilical 12mm port site, after slight extension of the fascial incision. The specimen size was 29.8g and 50x42x38mm nodular firm pale yellow mass with attached piece of gastric tissue, 32x8x3mm. The operative time was 75 minutes. Cardiorespiratory parameters and urine output remained adequate throughout the procedure.

Post-operative plan

*1.* *Post-Operative Review by Anaesthetist and Intensive Care Physician*

Following the laparoscopic resection, the patient was assessed in recovery by both the anesthetist and intensivist to evaluate her recovery. Given her stable haemodynamic status and the absence of immediate postoperative complications, it was decided that admission to the ward was more appropriate than to the ICU. This decision also minimised her exposure to invasive interventions and considered the increased risk of ICU-related delirium in older patients, particularly those with dementia or cognitive impairment.

2. Family Presence to Aid Familiarisation and Minimise Confusion

To support the patient's recovery, her family was permitted to stay overnight which helped reduce the likelihood of postoperative delirium and cognitive impairment, and facilitated the patient’s adjustment to post-operative care. The familiar environment of the general ward with continuity of care, coupled with family support, was deemed to offer a better setting for her cognitive function.

3. Minimisation of Lines/Tubes

Given the patient’s frailty and the risk of complications associated with prolonged use of invasive devices, efforts were made to limit the need for lines. Indwelling urinary catheter (IDC) was removed on day 1 post-operatively to reduce the risk of urinary tract infection and to facilitate early mobilisation. The patient did not require an NGT post-operatively, as she was able to tolerate oral intake relatively early on day 1 after the procedure.

4. Analgesia Strategy

Paracetamol was administered regularly as the primary analgesic. Opioids were used sparingly for breakthrough pain, with the goal of limiting their duration.

5. Early Review and Mobilisation with Physiotherapy

The physiotherapy team was involved since day 1 of the surgery and aided with mobilisation. She was given guidance on mechanical venous thromboembolism prophylaxis for early post-discharge period.

6. Feeding Plan/Nutritional Support

The patient was started on a clear liquid diet post-operatively and progressed to lactose-free light diet as tolerated on day 1 post-operatively. Given her history of weight loss and iron deficiency anaemia, a dietitian was consulted who provided education on high energy/high protein diet and oral energy fibre supplements to assist in her recovery. Her blood glucose levels were closely monitored.

7. Early Discharge Home with Family Support and Short-Interval Follow-Up

Given the patient’s stable recovery, functional baseline and adequate family support, she was deemed safe for discharge home day 1 post-operatively. A follow-up review with the surgeon was conducted at two- and six-week post-procedure. Her recovery was uneventful without major complications, such as cardiovascular events or wound infection.

**Figure 3:**
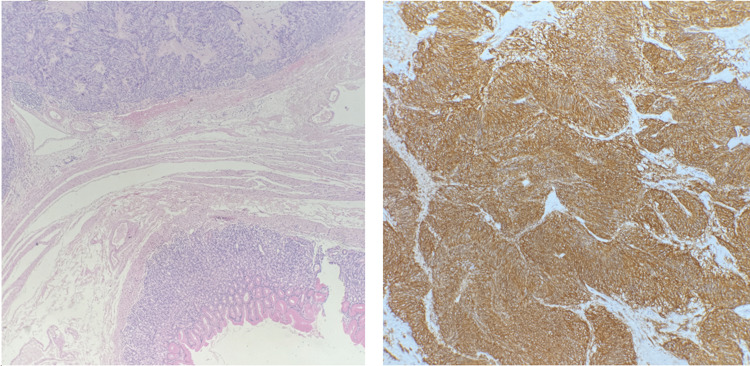
(Left) H&E at 40x magnification showing a well circumscribed, unencapsulated lesion with overlying gastric mucosa. The lesion is centred within the muscularis propria and not emerging from the overlying mucosa. (Right) DOG1 IHC at 200x power showing diffuse cytoplasmic positive staining.

Histopathological assessment confirmed a 50 mm GIST with intralesional haemorrhage and low mitotic index (<5%), resected with clear margins. The tumour cells are positive for DOG1 and DC117 and negative for SMA and S100. Ki67 is <5% (see Figure 3 below).

She remains well past 15 months post-surgery with no recurrence of symptoms. Given her advanced age, no follow-up upper GI endoscopy and/or CT-imaging has been scheduled.

## Discussion

This case report highlights the successful application of laparoscopic surgery and tailored peri-operative management in an exceptionally elderly patient. As life expectancy continues to rise - particularly in developed nations like Australia - an increasing number of centenarians are likely to present for surgical intervention [2]. However, the body of research addressing the perioperative experiences and outcomes of elderly patients remains limited, highlighting a critical gap in clinical evidence.

Surgical interventions in centenarians present considerable difficulties due to age-related physiological decline, comorbidities, and limitations in functional status. These factors often complicate the management process and may influence recovery outcomes. This necessitates a more nuanced approach to surgical decision-making, emphasizing individualised assessments that consider each patient’s overall health status and allow for a focused allocation of hospital resources.

Scheduling the procedure electively likely contributed to achieving a favourable outcome as elective surgery in patients over 90 years old is associated with lower mortality and improved two-year survival rates compared to emergency surgery [7,8]. The difference in outcomes between elective and emergency surgeries for patients aged over 90 years is particularly striking, with significantly higher 90-day mortality rates observed in emergency cases (90-day mortality rate 5.2% in elective vs 19.4% in emergency) [7]. Likewise, two-year survival of patients undergoing non-oncological surgery is 72.7% in elective setting, compared with 40.6% in emergency situations (p=0.0005) [8].

Elderly patients undergoing surgery are particularly vulnerable to complications, including delirium (10-30%), pneumonia or respiratory failure requiring intensive care unit support (5-15%), myocardial ischemia or arrhythmia (5-10%), DVT (15-30%) or fatal PE (0.2-0.9%), renal impairment (0.8-22.4%), and functional decline (10-30%) [9-14]. Surgery-specific complications include post-operative bleeding (3-7%) [15] and wound infection (1-18%) [16].

Multiple factors impact overall surgical outcomes in very elderly patients, with careful consideration given to patient’s age, frailty, and comorbid conditions. Multidisciplinary collaboration and thorough perioperative care are crucial in achieving optimal outcomes, a key factor in the positive outcome of this case. Coordinated efforts among surgeons, anaesthetists, and allied health professionals were critical in optimizing perioperative care and minimising complications. Collaborative care models, such as those integrating orthopaedic and geriatric physician services, have already demonstrated significant improvements in mortality rates, incidence of delirium and functional status, and reduced cost compared to standard care. Implementation of comprehensive geriatrician assessments has proven effective in addressing the specific needs of elderly patients, where both surgeons and physicians share responsibility of the care of the patient leading to reduced hospital length of stay and improved postoperative outcomes [17]. Early involvement of allied health professionals also improves patient care, especially in nutrition and mobilisation, leading to reduced overall postoperative complications and length of hospital stay [18]. In this current case, physiotherapists and dieticians provided advice and education regarding breathing exercises, preventive measures for deep venous thrombosis (DVT) and guidance on optimal high-protein, high-energy light diet.

Prolonged operative time is related to the likelihood of various postoperative complications, with risks often rising with age [19]. Prioritising thorough preoperative planning, experienced surgical team capable of delivering similar outcome under challenging conditions and low-pressure pneumoperitoneum, as well as attentive circulating nurses can help reduce operative time.

Post-operative pain management is a critical part of the recovery process. A multimodal analgesia approach was used to manage the patient’s pain, aiming to minimise opioid use and its associated risks (e.g., sedation, constipation, delirium).

Age alone is not a criterion for intensive care unit (ICU) admission. This ethos is supported by Demoule et al., with illness severity being a much more relevant predictor of need for ICU-level care. Patients aged >90 years undergoing intermediate-risk procedures can be managed safely and effectively in a general ward setting, given suitable patient selection and effective intraoperative haemodynamic management [20]. In a prospective study done by Ouimet et al., older patients, particularly those with dementia or cognitive impairment, are at higher risk of developing ICU-related delirium [21]. In the presented case, permitting the patient’s next-of-kin to stay overnight with the patient on the ward and followed up by prompt discharge home to reintegrate a familiar living environment, assisted to avoid post-operative confusion.

Understanding the risks faced by a very elderly cohort of patients allows for proactive strategies for mitigation. Key considerations include: minimising operative time by involving experienced surgical team, maintaining a constant low-pressure pneumoperitoneum (8 mmHg) to minimise the impact on cardiorespiratory parameters (venous return, ventilation pressures, etc.), which in turn contributed to reduction in postoperative pain, optimizing anaesthesia with utilisation of multimodal analgesia, multidisciplinary involvement of medical and allied health staff, early ambulation protocol, early discharge planning and rehabilitation.

Even though surgical interventions in patients aged over one hundred years pose unique challenges, the decision-making process should be individualised, considering general health status, disease characteristics and potential benefits of the treatment.

## Conclusions

With advancements in minimally invasive surgical techniques and customized perioperative care, age should not be a limiting factor in considering surgical intervention for many operable conditions, including GISTs. This case underscores the importance of individualized decision-making and tailored management, with a focus on multidisciplinary care, minimizing operative time, optimising anaesthesia through multimodal analgesia, and ensuring early ambulation, discharge planning, and rehabilitation. Such strategies are crucial for enhancing recovery, optimizing outcomes, and improving quality of life.

This case report also serves as a reminder that surgical interventions for centenarians can be both feasible and effective when appropriate care protocols are followed. It highlights the importance of challenging traditional assumptions that age alone should preclude surgical treatment and emphasizes the need for a comprehensive, patient-centered approach. The positive outcome in this case further reinforces the value of taking an individualized approach to geriatric surgery, offering a path forward for other centenarians with similar conditions. However, it is important to acknowledge that this case report details a single instance, and further research and documented case studies are needed to expand our understanding and refine treatment strategies for this rare demographic. Prospective studies and clinical trials exploring the outcomes of surgery in centenarians, particularly those involving minimally invasive techniques and targeted perioperative care, are crucial in advancing our knowledge. Additionally, updated guidelines in geriatric surgery, informed by emerging evidence, would be invaluable in guiding clinicians in providing the best care for this aging population.
